# Retracted: *Allium cepa* L. and Quercetin Inhibit RANKL/*Porphyromonas gingivalis* LPS-Induced Osteoclastogenesis by Downregulating NF-*κ*B Signaling Pathway

**DOI:** 10.1155/2019/7906103

**Published:** 2019-08-14

**Authors:** Evidence-Based Complementary and Alternative Medicine

Evidence-Based Complementary and Alternative Medicine has retracted the article titled “*Allium cepa* L. and Quercetin Inhibit RANKL/*Porphyromonas gingivalis* LPS-Induced Osteoclastogenesis by Downregulating NF-*κ*B Signaling Pathway” [1]. The article was found to include duplicate Western blots in Figure 5 representing different experiments as follows:

(i) In Figure 5(a), the last two bands in the last lane (*β*-actin) of the first image appear to be the same as the first two bands in the last lane (*β*-actin) of the third image.

(ii) In Figure 5(b), the first two bands in the second lane (I*κ*B*α*-p) of the first image appear to be the same as the last two bands in the first lane (I*κ*B*α*) of the second image.

(iii) In Figure 5(b), the first two bands in the first lane (I*κ*B*α*) of the second image appear to be the same as the last two bands in the first lane (I*κ*B*α*-p) of the third image.

(iv) In Figure 5(b), the first band in the second lane (I*κ*B*α*-p) of the first image appears to be the same as the second band in the first lane (I*κ*B*α*) of the second image and the last band in the first lane (I*κ*B*α*-p) of the third image.

(v) In Figure 5(b), the last lane (*β*-actin) of the first image appears to be the same as the last lane (*β*-actin) of the third image.

This concern was raised on PubPeer and confirmed by the Editorial Board.

Therefore, we asked the authors to explain this and to provide us with the original uncropped and unadjusted images for Figures 1(a), 3(a), 4(a), and 5, and the underlying raw data for Figures 1(b), 2, 3(b)–3(d), 4(b), and 5 and Table 1. The authors were unable to provide the underlying blots, because the work was done several years ago and the supervisor of the Ph.D. student, Tatiane Oliveira, in Canada, Dr. Getulio Nogueira-Filho, left his faculty position at the University of Toronto in 2014 and all the equipment was reallocated and no backup was done. However, the authors provided data related to the quantification of the blots (which agreed with the data) and stated that a mistake might have occurred while they were editing the figures, but it does not represent an error in the analysis and quantification of the data represented in the graphics of Figure 5.
